# Predictive factors of response to interferon in chronic HBV hepatitis

**DOI:** 10.1186/1471-2334-14-S7-P55

**Published:** 2014-10-15

**Authors:** Cristina Popescu, Alina Lobodan, Gabriel Adrian Popescu, Mihaela Rădulescu, Violeta Molagic, Anca Negru, Daniela Munteanu, Iulia Caragea, Angelica Teniță, Victoria Aramă

**Affiliations:** 1 National Institute for Infectious Diseases "Prof. Dr. Matei Balş", Bucharest, Romania; 2 Carol Davila University of Medicine and Pharmacy, Bucharest, Romania

## Background

It is well known that the virological response after pegylated interferon (IFN) treatment in chronic HBV hepatitis is less than 50%. The main advantage of IFN is the finite duration of therapy. A response guided therapy in this situation would be very important in order to recognize earlier the patients with poor response. Unfortunately, Romanian guidelines for HBV hepatitis treatment do not include a response guided therapy and IFN-based regimens are recommended for one year. Objective: To analyze the factors correlated with the virological response to IFN in chronic HBV hepatitis.

## Methods

We made a retrospective analysis of the HBV chronically infected patients treated with IFN, monitored in Third Department of Matei Bals Institute. Patients were divided in two groups: group 1, with virological response and group 2, without virological response. The virological response to IFN is defined as viral load <2000 IU/mL after one year of follow-up (according to EASL guideline). The inclusion criteria in our study: HBV infected patients treated one year with IFN who have finished the therapy for more than one year.

## Results

Fifty-six patients met the inclusion criteria. 23 patients achieved virological response (group 1) and 33 patients didn’t respond to IFN and were subsequently treated with entecavir (group 2). Mean age was similar in both groups: 38.4 vs. 38.54 year-old. Sex ratio was M:F=1:1.8 in group 1 and M:F=3.125:1 in group 2; therefore, male gender represents a risk factor for non-response to IFN – RR=2.18 (1.21;3.94). The rate of positive HBeAg was 21.73% in group 1 and 24.24% in group 2. The presence of HBeAg was not statistically correlated with poor response to IFN: RR=0.92 (0.42; 1.99), but the HBe seroconversion during IFN therapy was a very good predictor for response: RR=6 (1; 35.9), p=0.005. The starting level of viral load below 106 IU/mL was considered a predictive factor for a good response to IFN – RR=1.43 (1.02; 2.27), p=0.04. Increased ALT was not associated with a good response to IFN (despite literature data), RR=1.16 (0.81; 1.66), p=0.55. We point out that our patients were not systematically evaluated regarding the viral load level after three and six months of therapy.

## Conclusion

The use of IFN in selected patients can improve the virological response. Female gender, HBe seroconversion during therapy and the viral load below 106 represent good predictors to response. Positive HBeAg and increased ALT at baseline were not associated with virological response.

